# Intensity-dependent lipidomic dynamic regulation following acute swimming exercise

**DOI:** 10.1038/s41598-026-39013-5

**Published:** 2026-02-10

**Authors:** Jiayu Qian, Baile Wu, Zhongxun Ren, Chunxue Tang, Zihan Fan, YanYan Zhang, Lijun Shi

**Affiliations:** 1 Department of Exercise Physiology, Beijing Sport University, Beijing, 100084 P. R. China; 2 Laboratory of Sports Stress and Adaptation of General Administration of Sport, Beijing Sport University, Beijing, China; 3 Key Laboratory of Sports and Physical Health of Ministry of Education, Beijing Sport University, Beijing, China

**Keywords:** Acute swimming exercise, High-intensity interval training (HIIT), Moderate-intensity continuous training (MICT), Lipidomics, Lipid metabolism, Biochemistry, Biomarkers, Physiology

## Abstract

Exercise intensity critically determines health benefits, yet the underlying molecular mechanisms remain incompletely characterized. This study systematically compared the effects of high-intensity interval training (HIIT) versus moderate-intensity continuous training (MICT) swimming on serum lipidomics. In a randomized controlled trial (ChiCTR2400089036, registered on 30/08/2024, *n* = 42 healthy students), blood samples were collected at baseline, 0-, 15-, and 30-minutes post-exercise, followed by comprehensive lipidomics analysis. HIIT swimming induced 1.49- to 2.87-fold more extensive serum lipid downregulation than MICT, despite matched energy expenditure. We identified five robust intensity-dependent biomarkers: PC32:2, LPA18:2, and three 18:2-containing triacylglycerols (TAGs). Clustering analysis further revealed three distinct hierarchical patterns of lipid dynamic changes. Structurally, HIIT preferentially mobilized shorter-chain, saturated TAGs, and post-exercise lipid recovery dynamics were also intensity-dependent. The recurrent identification of linoleic acid (18:2)-enriched lipids, which negatively correlated with energy metabolites, suggests coordinated substrate channeling toward inflammatory eicosanoid pathways. These findings advance the mechanistic understanding of exercise intensity benefits and provide molecular evidence for intensity-stratified exercise prescription.

## Introduction

Regular physical activity significantly reduces chronic disease risk and improves cognitive function, mood, and quality of life^1,2^. Exercise intensity represents a critical determinant of these health benefits^3^, yet the molecular mechanisms underlying intensity-dependent adaptations remain incompletely characterized. High-intensity interval training (HIIT) and moderate-intensity continuous training (MICT) demonstrate fundamentally different metabolic strategies. HIIT predominantly activating fast glycolytic pathways with enhanced post-exercise excess oxygen consumption (EPOC)^4^, while MICT relies on sustained aerobic metabolic pathways^5^. Although both modalities improve cardiorespiratory fitness and insulin sensitivity, their underlying molecular mechanisms differ fundamentally^6,7^,suggesting distinct metabolic regulatory networks.

Swimming, with its unique aquatic environment properties including high resistance and rapid heat transfer, provides an ideal model for studying intensity-dependent exercise responses^8–11^. Elucidating these mechanisms requires advanced analytical methods. Traditional lipid analysis methods are inadequate for this purpose, as they are limited to basic indicators like total cholesterol and triglycerides and fail to reflect the fine regulation of lipid metabolism^12^. The human lipidome contains over 3,000 different molecules, with diverse biological functions including membrane structure, energy storage, and cellular signaling^13,14^. Lipidomics provides the necessary analytical power to simultaneously detect hundreds to thousands of lipid molecules, enabling comprehensive characterization of exercise-induced metabolic changes^15^. While recent studies have utilized lipidomics to resolve chronic training effects on circulating lipid profiles^16,17^, investigations of acute exercise responses, particularly swimming at varying intensities, remain scarce^18^. Based on this background, this study aimed to systematically compare the differential modulatory effects of HIIT and MICT swimming exercise on serum lipid profiles using high-throughput lipidomics technology, providing molecular-level foundations for developing precision swimming prescriptions and intensity-specific nutritional interventions.

## Materials and methods

### Study design and swimming exercise protocol

This study utilized a randomized controlled trial design. Participant inclusion and exclusion criteria have been described previously^19^. All participants were healthy, recreationally active university students with no chronic diseases, cardiovascular conditions, or metabolic disorders. None were competitive athletes or engaged in structured training. All participants could complete 50-meter freestyle swimming. Baseline characteristics are presented in Table 1. Forty-two participants were randomly assigned to the HIIT (*n* = 21) or MICT (*n* = 21) group. Both groups completed a one-week adaptation period before undergoing the formal swimming test in a fasted state (10–12 h overnight fast) on the third morning.

After a 10-minute warm-up, the HIIT group performed repeated cycles of 50-meter maximal sprints with 2-minute passive recovery for 30 min, while the MICT group completed 30 min of moderate-intensity swimming (70–80% HRmax). Heart rate was continuously monitored using the BHT-TEAM telemetry system (BoHaoTong, China), and RPE was assessed post-exercise using the Borg 6–20 scale.

The study was approved by BSU-IRB (protocol: 2022188 H) in accordance with the Declaration of Helsinki and was prospective registered in the Chinese Clinical Trial Registry (ChiCTR2400089036, first registered 30/08/2024). All participants provided written informed consent. Swimming performance data are shown in Table 2.

**Table 1:** Baseline participant Characteristics.

Sex (male; female)	MICT (*n* = 21) 10;11	HIIT (*n* = 21) 10;11	MICT Vs. HIIT (*P* value) -
Anthropometric measures			
Height (m)	168.0 (8.7)	169.4 (10.2)	0.489
Body mass (kg)	62.9 (55.7, 72.3)	66.6 (53.9, 75.8)	0.624
Age (years)	21.0 (20.0, 25.0)	22.0 (20.0, 24.0)	0.732
BMI (kg/m²) Body fat (%) Waist-to-Hip Ratio (WHR)	22.2 (20.4, 24.1) 27.0 (5.7) 0.79 (0.06)	22.6 (20.4, 24.4) 26.9 (6.6) 0.8 (0.08)	0.782 0.734 0.494
Functional physiological measures			
Resting Heart Rate (beats/min)	67.0 (58.5, 71.5)	61.0 (57.0, 68.5)	0.194
METs (MET-minutes/week)	898 (435, 1824)	989 (680, 2290)	0.649
Screening test			
Duration of 50 m sprint swimming (s)	71.0 (70.0 ~ 92.0)	71.0 (61.0 ~ 80.0)	0.130
Heart rate of 50 m sprint swimming (bpm)	162.2 (14.0)	163.7 (15.8)	0.860
*RPE of 50 m sprint swimming (Borg 6–20)	15 (14.0 ~ 17.0)	16.0 (15.0 ~ 17.0)	0.287

*RPE: Rate of Perceived Exertion. Values shown as mean (SD) for normal distributions and median (P25, P75) for non-normal distributions. Mann-Whitney U tests were used for between-group comparisons.

**Table 2:** Swimming performance assessment Results.

	MICT	HIIT	*P* value
Total swimming duration (min)	30.0 (30.0, 31.0)	29.0 (28.0, 31.0)	0.053
Net swimming duration (min)	30.0 (30.0, 31.0)	12.0 (11.0, 12.5)	< 0.001
50m sprint duration in HIIT actual testing (s)	-	70.5 (8.9)	-
Total swimming distance (m)	906.4 (131.5)	492.9 (32.7)	< 0.001
^#^Total energy expenditure (kcal)	249.4 (47.7)	270.8 (62.8)	0.204
Average swimming speed (m/s)	0.50 (0.07)	0.71 (0.09)	< 0.001
Average Heart Rate in swimming (bpm)	138.0 (9.4)	143.9 (11.3)	0.082
Maximum Heart Rate in swimming (bpm)	155.4 (5.6)	182.8 (5.0)	< 0.001
*RPE of swimming (Borg 6-20)	12 (11,14)	17 (15,180	< 0.001

*RPE: Rate of Perceived Exertion. Values are presented as mean (SD) for normally distributed data and median (P25, P75) for non-normally distributed data. Between-group comparisons were analyzed using non-parametric Mann-Whitney U tests to determine p values.^#^Total energy expenditure values during swimming exercise were automatically calculated using the BHT-TEAM wearable telemetry heart rate monitoring system (BoHaoTong, China).

### Blood collection and serum preparation

Participants provided fasting morning blood samples from the cubital vein at the initial timepoint. Following each swimming session, additional venous samples were obtained within 2 min of pool exit, with subsequent collections at 15-minute and 30-minute recovery intervals. All collections utilized BD Vacutainer tubes (red-top, anticoagulant-free). Samples underwent coagulation for 45 min under ambient conditions prior to centrifugation (1200 g, 10 min, 4℃) using a refrigerated centrifuge. Resulting serum was aliquoted into pre-labeled 1.5mL cryovials and maintained at -80℃ pending analysis. All sample underwent only one freeze-thaw cycle.

### Lipid and metabolite extraction

For lipid profiling, 50-µL serum aliquots underwent biphasic extraction using a modified Bligh-Dyer protocol adapted from established methodology^20^. The extraction procedure commenced with the addition of 750 µL chloroform: methanol solution (1:2, v/v) to each sample, followed by orbital agitation (1500 rpm) for 30 min at 4℃ to facilitate lipid solubilization. Phase partitioning was then induced through sequential addition of deionized water (350 µL) and chloroform (250 µL). Following centrifugal separation, the lower lipid-rich organic layer was carefully aspirated and transferred to fresh microcentrifuge tubes. To maximize lipid recovery, the residual aqueous phase underwent a second extraction cycle with an additional 450 µL of chloroform. The combined organic extracts were consolidated in a single tube and concentrated to dryness using a SpeedVac vacuum concentrator (OH mode setting). Dried lipid residues were maintained at -80℃ until instrumental analysis.

For metabolite profiling, frozen serum samples underwent controlled thawing at 4 °C with gentle vortex mixing, followed by clarification centrifugation to remove particulate matter. A 100-µL aliquot of clarified serum was then combined with 400 µL of ice-chilled methanol containing 0.28 mM phenylhydrazine to facilitate chemical derivatization of α-ketoacid species^21^. Following vigorous vortex mixing, samples were incubated at -20 °C for 30 min to allow complete derivatization. The reaction mixture was then subjected to high-speed centrifugation (12,000 rpm, 10 min, 4 °C) to pellet any precipitated proteins. The clarified supernatant was quantitatively transferred to clean 1.5 mL microcentrifuge tubes and concentrated to dryness via SpeedVac vacuum centrifugation. For instrumental analysis, dried metabolite extracts were reconstituted in 100 µL of 5% acetonitrile solution with thorough vortex mixing, followed by a final clarification spin (12,000 rpm, 10 min, 4 °C) to remove any remaining particulates.

### LC-MS-Based targeted lipidomics and metabolomics profiling of serum

#### Serum targeted lipidomics profiling

Serum lipidomic profiling was performed using a targeted multiple reaction monitoring (MRM) approach on a Sciex TRIPLE QUAD 4500 MD tandem mass spectrometry system coupled with an ultra-performance liquid chromatography (UPLC) system^22^. Chromatographic separation was achieved on a TUP-HB silica column (3 μm, 150 × 2.1 mm i.d.) operated at 25 °C. The mobile phase consisted of (A) chloroform: methanol: ammonia (89.5:10:0.5, v/v/v) and (B) chloroform: methanol: ammonia: water (55:39:0.5:5.5, v/v/v/v) delivered at a flow rate of 0.3 mL/min. The injection volume was 5 µL. The gradient elution program was as follows: mobile phase A was maintained at 95% for 5 min, then linearly decreased to 60% over 7 min and held for 4 min, further decreased to 30% and held for 15 min, and finally returned to initial conditions and equilibrated for 5 min. The internal standard cocktail included: d9-PC32:0(16:0/16:0), PE 34:0, dic8-PI, d31-PS, C17:0-PA, DMPG, CL-14:0, C14-BMP, C12-SL, C17-LPC, C17-LPE, C17:1-LPI, C17:0-LPA, C17:1-LPS, C17-Cer, C12-SM, d17:1-S1P, d17:1-Sph, C8-GluCer, C8-LacCer, and Gb3-d18:1/17:0 (all sourced from Avanti Polar Lipids); GM3-d18:1/18:0-d3 (Matreya LLC); d31-16:0 for free fatty acid quantitation (Sigma-Aldrich); and d6-CE 18:0 plus TAG(16:0)3-d5 (CDN Isotopes). Mass spectrometric detection was performed in positive electrospray ionization (ESI+) mode with the following parameters: curtain gas, 20 psi; ion spray voltage, 5500 V; source temperature, 400 °C; ion source gas 1 (nebulizer gas), 35 psi; ion source gas 2 (heater gas), 35 psi. Data acquisition was conducted in MRM mode monitoring 608 lipid species across multiple lipid classes. Peak integration and quantification were performed using MultiQuant software (version 3.0.3, Sciex; https://sciex.com/products/software/multiquant-software).

#### Serum targeted metabolomics profiling

Targeted metabolomic analysis of 8 energy metabolism-related metabolites was performed using the same LC-MS/MS system. Chromatographic separation was achieved on an ACQUITY UPLC HSS T3 column (1.8 μm, 3.0 × 100 mm; Waters, Dublin, Ireland) maintained at 40 °C. The mobile phase consisted of (A) 0.1% formic acid in water (v/v) and (B) 100% acetonitrile delivered at a flow rate of 0.3 mL/min. The injection volume was 1 µL. The gradient elution program was as follows: 0–1.0 min, 2% B; 1.0–6.0 min, 2–42% B (linear gradient); 6.0–8.0 min, 42–65% B; 8.0–10.0 min, 65–76% B; 10.0–11.0 min, 76–100% B; 11.0–14.0 min, 100% B (isocratic hold). Mass spectrometric detection was performed in negative electrospray ionization (ESI-) mode with the following parameters: curtain gas, 35 psi; ion spray voltage, -4500 V; source temperature, 450 °C; ion source gas 1, 50 psi; ion source gas 2, 50 psi. Data acquisition was conducted in MRM mode with optimized compound-specific parameters. Peak integration and quantification were performed using MultiQuant software (version 3.0.3, Sciex; https://sciex.com/products/software/multiquant-software).

### Statistical analysis and data visualization

Statistical power analysis was conducted using G*Power software (version 3.1.9.7; https://www.psychologie.hhu.de/arbeitsgruppen/allgemeine-psychologie-und-arbeitspsychologie/gpower), which determined that a minimum of 18 participants per group would be required to detect a medium effect size (Cohen’s d = 0.6) with 80% statistical power and α = 0.05. Raw lipidomic and metabolomic datasets were processed using R statistical environment (v4.4.1), with final visualization generated in Adobe Illustrator 2024. Data preprocessing included quantile-based normalization (preprocessCore package, v1.66.0) and z-score standardization (scales package, v1.3.0) to minimize technical variability.

The analytical strategy employed a multi-tiered approach centered on log_2_-fold change (log_2_FC) values. For each lipid and metabolite species, log_2_FC was calculated at each post-exercise timepoint (0, 15, and 30 min) relative to baseline measurements. Baseline demographic and physiological characteristics were compared between exercise intensity groups using independent samples t-tests (Tables 1 and 2).

**Fig. 1:**
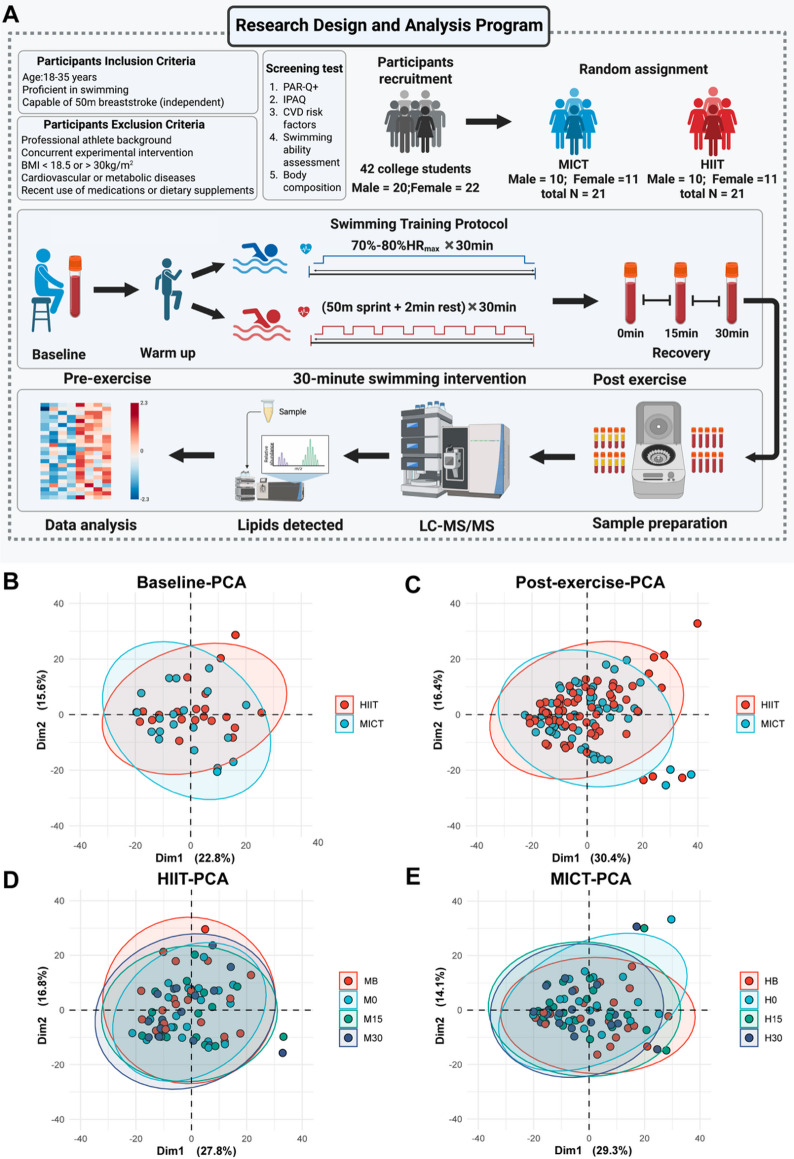
Study design and serum lipidomics profiling. (**A**) Experimental workflow showing participant screening, swimming protocol, sample collection, and lipidomics analysis. (**B**) PCA of baseline lipidomic profiles by intensity (608 lipid species). (**C**) PCA of combined post-exercise profiles (0-, 15-, 30-min) by intensity (608 lipid species). (**D-E**) PCA of temporal lipidomic trajectories for HIIT (**D**) and MICT (**E**) (608 lipid species).

**Fig. 2:**
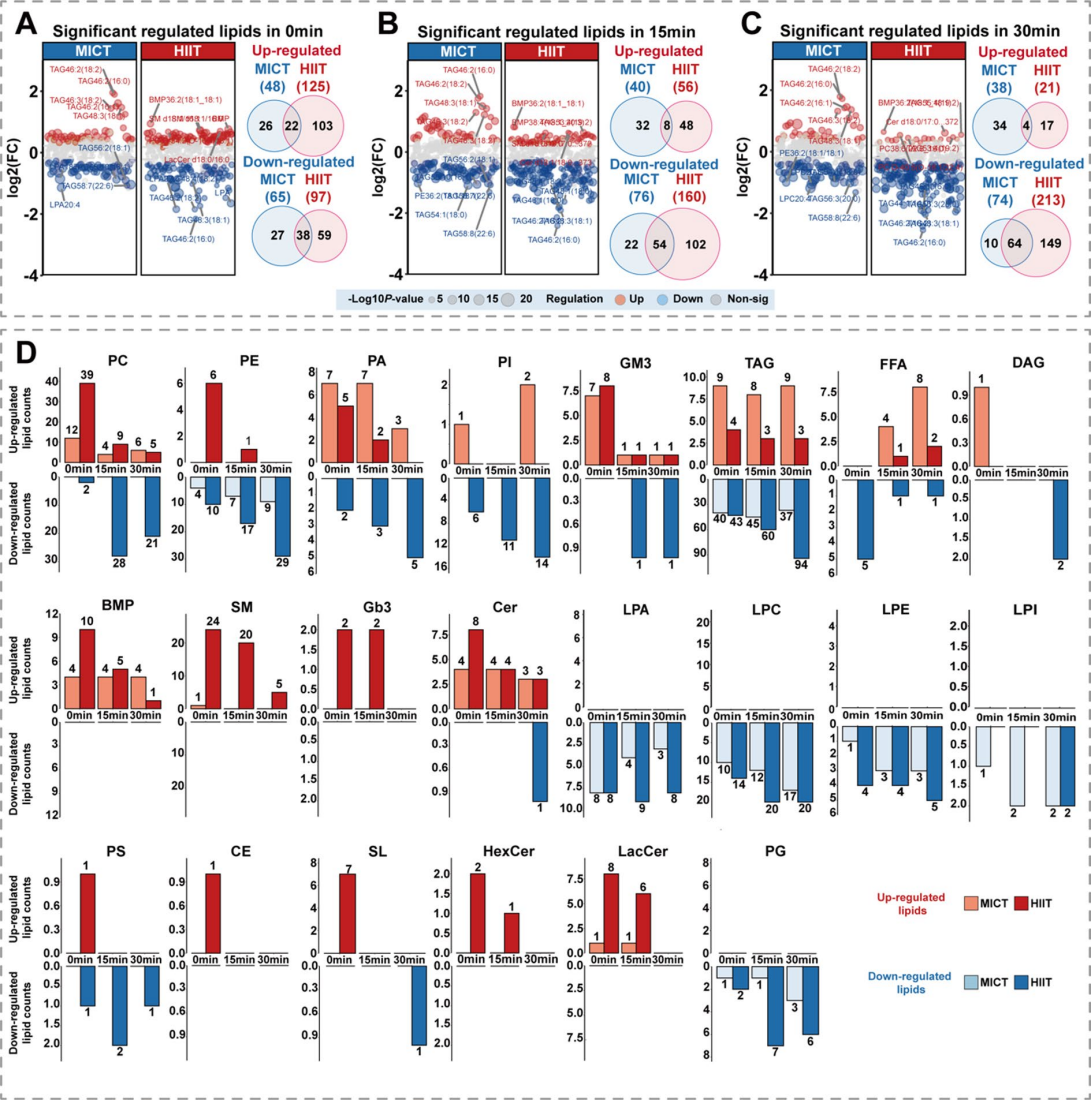
Overall characteristics and intensity differences of the serum lipidomic following acute swimming exercise. (**A-C**) Volcano plots of significantly regulated lipids at 0-, 15-, and 30-min post-exercise between HIIT and MICT (red representing up-regulated; blue representing down-regulated). Venn diagrams illustrate the overlap and unique regulation patterns between the two exercise modalities. (**D**) Differential lipids count by lipid subclass and time point including Phosphatidylcholine (PC), Phosphatidylethanolamine (PE), Phosphatidic acid (PA), Phosphatidylinositol (PI), Monosialodihexosylganglioside (GM3), Triacylglycerol (TAG), Free fatty acids (FFA), Diacylglycerol (DAG), Bis(monoacylglycero)phosphate (BMP), Sphingomyelin (SM), Globotriaosylceramide (Gb3), Ceramide (Cer), Lysophosphatidic acid (LPA), Lysophosphatidylcholine (LPC), Lysophosphatidylethanolamine (LPE), Lysophosphatidylinositol (LPI), Phosphatidylserine (PS), Cholesteryl ester (CE), Sulfolipid (SL), Hexosylceramide (HexCer), Lactosylceramide (LacCer), and Phosphatidylglycerol (PG).

To visualize global metabolic response patterns, principal component analysis (PCA) was applied to the complete log_2_FC dataset, enabling assessment of intensity-related segregation in lipid perturbation profiles (Fig. 1). Exercise-induced changes at individual timepoints were evaluated using paired t-tests comparing post-exercise values against baseline. Lipid species exhibiting *P* < 0.05 with |log_2_FC| > 0.2 were classified as significantly perturbed (Fig. 2).

**Fig. 3:**
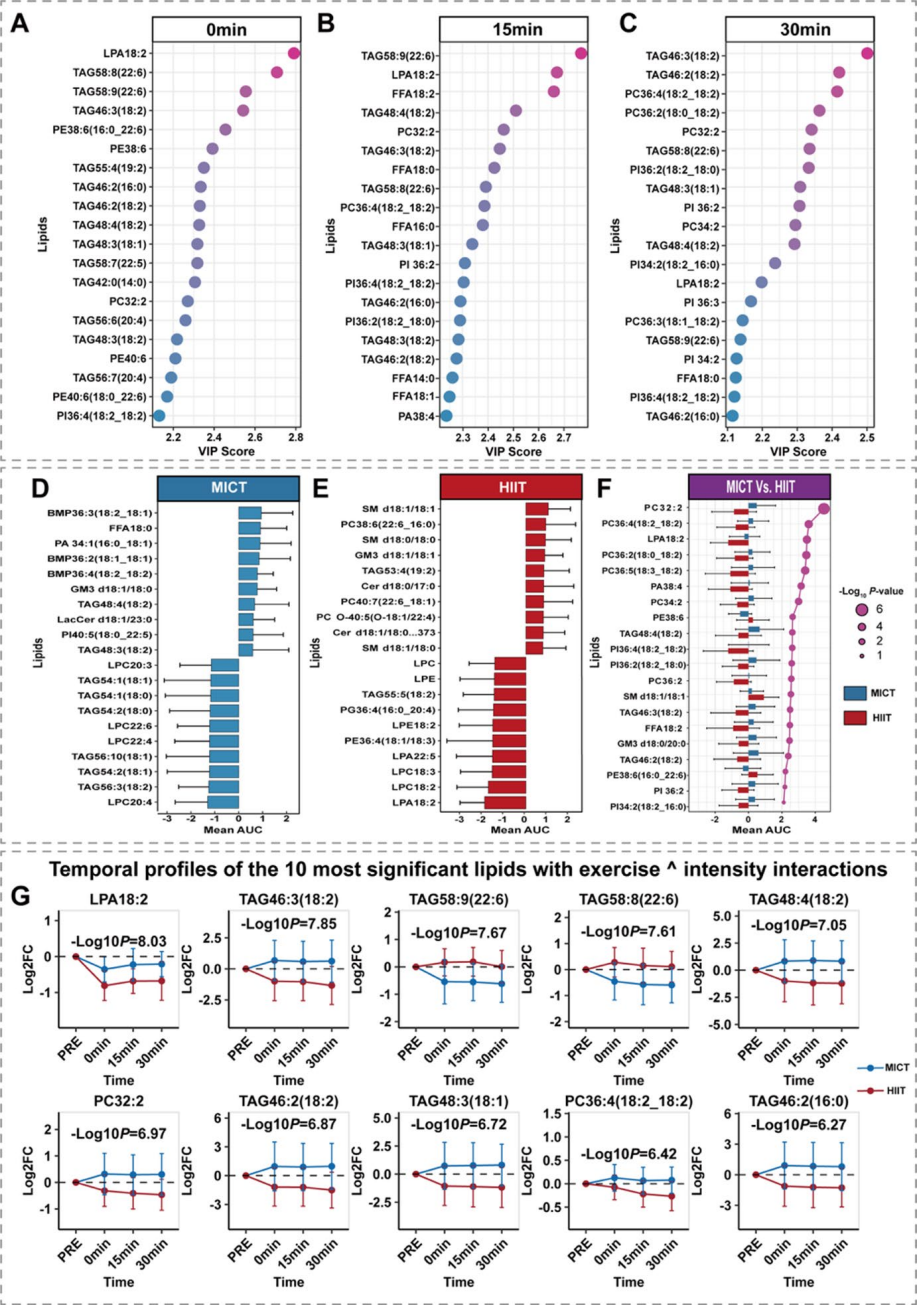
Identification of key intensity-differentiated lipids using multi-strategy analysis. (**A-C**) Top 20 lipids ranked by PLS-DA VIP scores at 0-, 15-, and 30-min post-exercise. (**D-E**) Top 20 most upregulated and downregulated lipids by mean AUC for MICT (**D**) and HIIT (**E**). (**F**) Top 20 lipids with greatest intensity differences in mean AUC; bar direction indicates higher cumulative response, point size represents -log10 adjusted P. (**G**) Temporal dynamics of top 20 lipids ranked by intensity main effect adjusted P-values using two-way ANOVA; blue lines represent MICT, red lines represent HIIT, displayed -log10 adjusted P corresponds to intensity main effect, data show mean ± SD.

To identify intensity-dependent lipid signatures, two-way repeated-measures analysis of variance (ANOVA) was conducted on log_2_FC datasets using the car R package (v3.1-2), with exercise intensity, time, and their interaction as factors (Fig. 3)^23^. Partial least squares discriminant analysis (PLS-DA; mixOmics v6.24.0) was performed with log2FC values of 608 lipid species as predictor variables (X) and group assignment (HIIT vs. MICT) as response variable (Y), with Variable Importance in Projection (VIP) scores used to rank discriminatory power. Mean area under the curve (AUC) was calculated to quantify cumulative perturbation magnitude (Fig. 3).

**Fig. 4:**
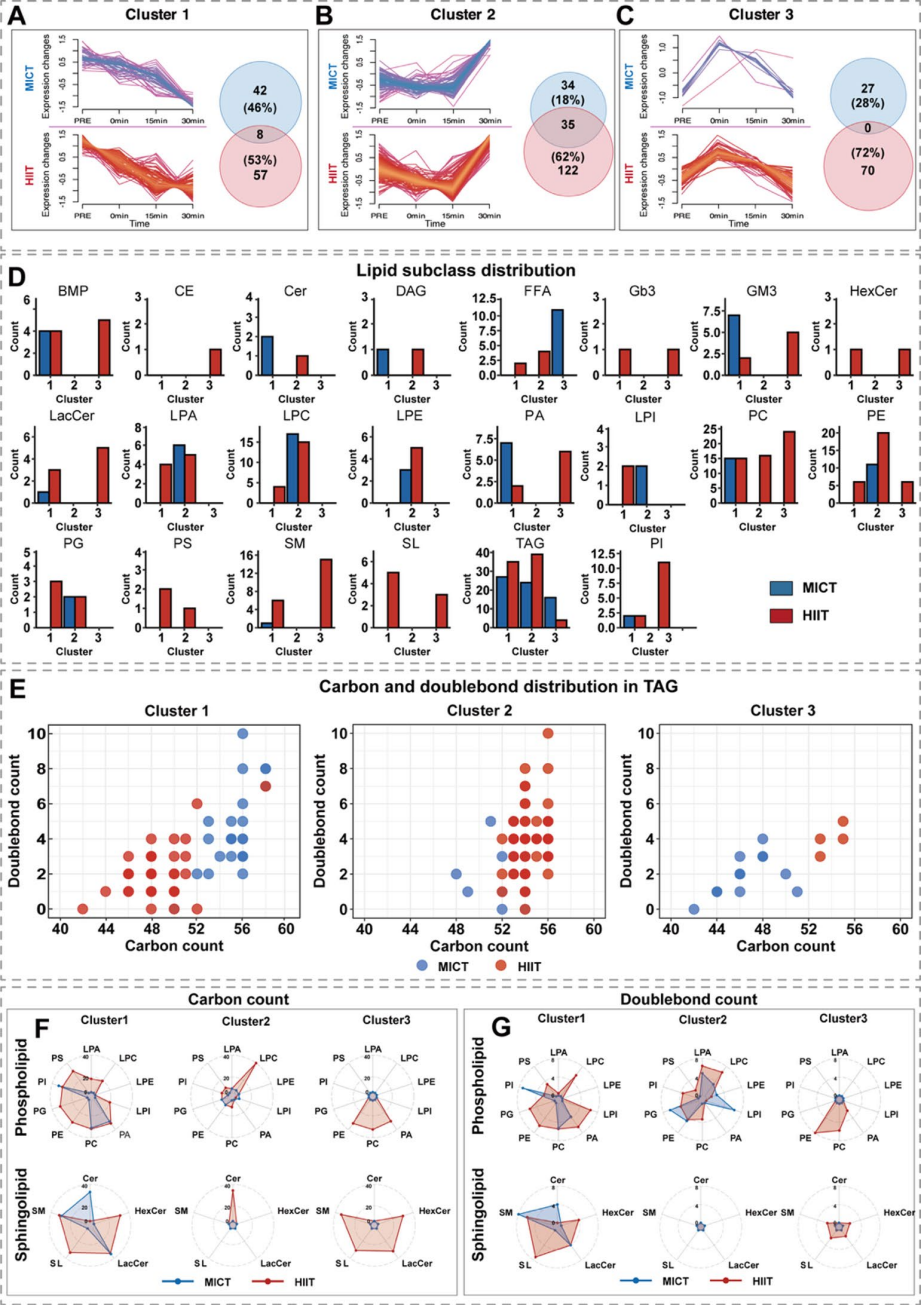
Intensity-dimorphic temporal dynamics and lipid chemical structure analysis in response to acute exercise. (**A-C**) Three major lipidomic patterns from fuzzy c-means clustering showing Cluster 1 (Sustained Downregulation), Cluster 2 (Recoverable Downregulation) and Cluster 3 (Recoverable Upregulation); line plots show temporal trends. (**D**) Intensity-specific distribution of lipid subclasses within temporal clusters. (**E-G**) Chemical structure analysis (carbon and doublebond) of lipids within three major lipid classes of TAG (**E**), phospholipids (**F**), and sphingolipids (**G**). In figures **E-G**, each point represents an individual significantly regulated lipid species (*P* < 0.05, |log_2_FC| > 0.2). X-axis shows total carbon number in fatty acid chains; Y-axis shows total number of double bonds. Points are color-coded by exercise intensity (blue = MICT, red = HIIT) and shaped by lipid subclass. The spatial distribution of points reveals intensity-specific preferences for particular chain lengths and saturation levels within each temporal cluster.

Fuzzy c-means clustering was implemented via the Mfuzz package (v2.62.0) to identify shared and distinct temporal response patterns across the complete log_2_FC dataset. The optimal clustering configuration (*n* = 3 clusters, fuzzification parameter m = 2.0) was selected based on cluster validity indices. Lipids meeting the significance criteria (*P* < 0.05, |log_2_FC| > 0.2) were included in the final analysis (Fig. 4).

Lipid structural characteristics, including fatty acid chain length and degree of unsaturation, were analyzed to identify intensity-dependent structural preferences. For TAGs, structural distributions were visualized using scatter plots (carbon number on x-axis, double bond count on y-axis), with each point representing a significantly regulated lipid species (*P* < 0.05, |log_2_FC| > 0.2). For phospholipids and sphingolipids, structural characteristics were displayed as radar charts, where each axis represents a lipid subclass and the radial distance indicates the mean carbon number (Fig. 4F) or mean double bond count (Fig. 4G). This approach enabled identification of intensity-specific structural selectivity across lipid classes (Fig. 4E-G).

**Fig. 5:**
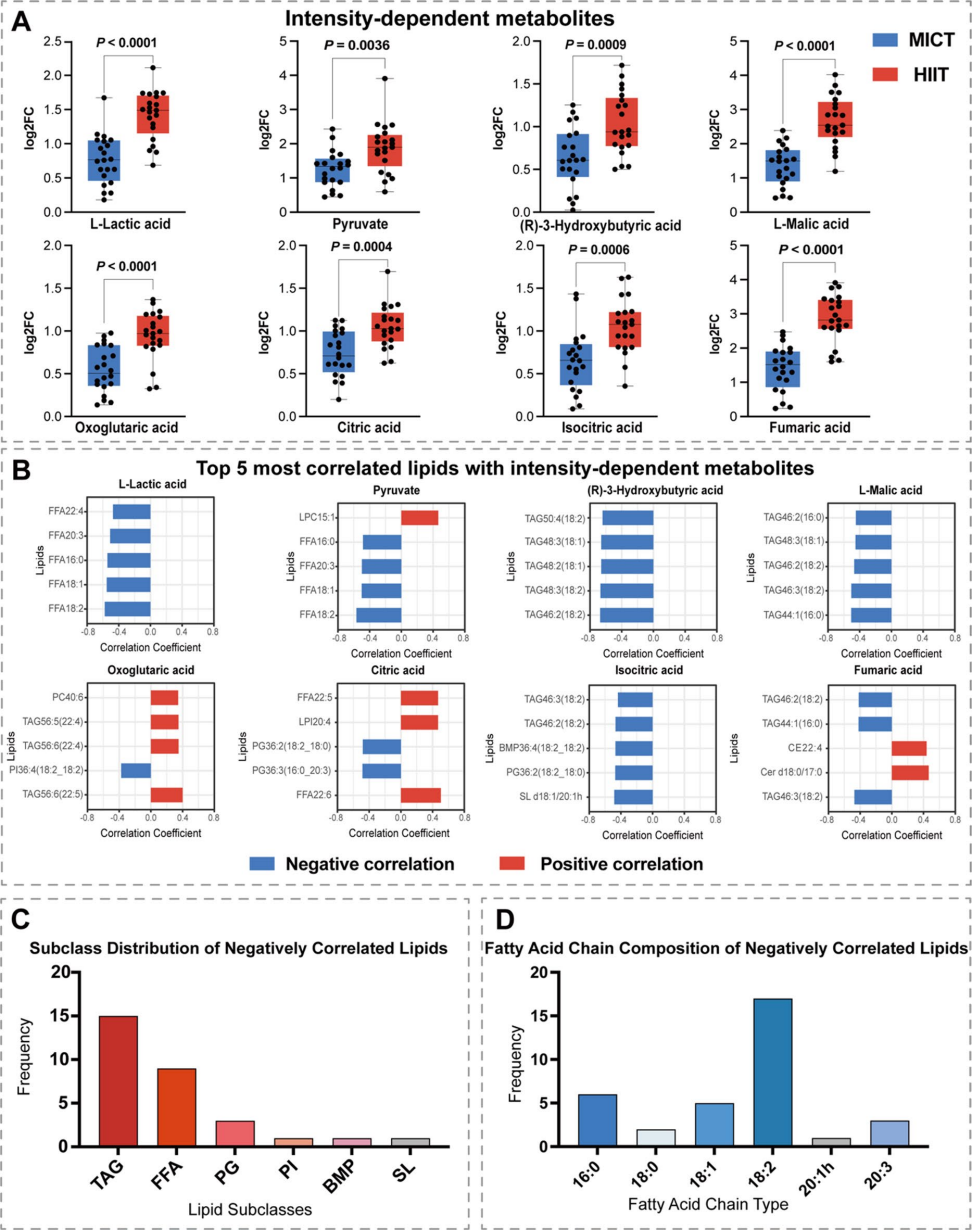
Intensity-dependent energy metabolites and their correlations with lipids. (**A**) Eight intensity-dependent energy metabolites identified by differential expression analysis between MICT and HIIT. (**B**) Top 5 most correlated lipids with each energy metabolites based on Pearson correlation coefficient. (**C**) Top 5 most correlated lipids with L-lactic acid (blue represents MICT and red represents HIIT; shaded areas show 95% confidence intervals). (**D**) Correlation heatmap of lipid subclasses and energy metabolites based on mean Pearson correlation coefficients.

To explore lipid-metabolite regulatory relationships, correlation analysis was performed between the maximum log_2_FC values of intensity-dependent metabolites and all lipid species using Spearman’s rank correlation coefficient. *P*-values were adjusted for multiple hypothesis testing using the Benjamini-Hochberg false discovery rate (FDR) method, with q < 0.05 considered statistically significant (Fig. 5).

## Results

### Baseline characterization and acute swimming exercise results

No statistical differences were observed between HIIT and MICT groups in morphometric indices including height, weight, body mass index (BMI), body fat percentage, and waist and hip measurements (*P* > 0.1). Similarly, age (*P* = 0.732), resting heart rate (*P* = 0.194), and metabolic equivalents (*P* = 0.649) showed no significant differences between groups. Total swimming duration (*P* = 0.053) and energy expenditure (*P* = 0.204) were similar between groups, but HIIT had significantly shorter net swimming time (*P* < 0.001), higher maximal heart rate (*P* < 0.001), and higher mean swimming speed (*P* < 0.001).

Principal component analysis was performed on the complete lipidomic dataset comprising 608 quantified lipid molecular species to assess global metabolic response patterns and group separation. Unsupervised PCA analysis showed no clear separation between groups or time points **(**Fig. 1B-E**)**. Baseline characteristics were consistent between groups, and intensity parameters were well-differentiated (Tables 1 and 2). Therefore, we employed more analytical strategies in subsequent studies and analyses to isolate and identify the true biological signals driven by exercise intensity.

### Panorama of serum lipidomic changes after acute swimming exercise of different intensities

We examined lipidome changes at three time points (0-, 15-, and 30-minutes post-exercise) after MICT and HIIT, with significantly altered lipids defined as those showing adjusted *P* < 0.05 and |log_2_FC| > 0.2 at any time point post-exercise relative to baseline.

The number of significantly down-regulated lipid molecules differed between HIIT and MICT across all post-exercise time points (Fig. 2A-C). At immediate, 15-minute, and 30-minute post-exercise periods, HIIT down-regulated 1.49-fold, 2.10-fold, and 2.87-fold more lipid molecules than MICT, respectively. Venn diagram analysis revealed that at 30 min post-exercise, HIIT down-regulated 15-fold more unique lipid molecules compared to MICT.

At the lipid subclass level **(**Fig. 2D**)**, HIIT down-regulated a greater number of lipid subclasses than MICT, and this increased during the post-exercise recovery period. Both groups exhibited differential up-regulation across subclasses. HIIT-induced up-regulation was highest immediately post-exercise and decreased thereafter, whereas MICT exhibited stable up-regulation throughout the recovery period. TAG showed the largest number of regulated species among all lipid subclasses. Additionally, CE and SL were exclusively regulated by HIIT at all time points, while no MICT-specific subclasses were identified.

### Intensity-dependent differential lipid screening

To identify exercise intensity-dependent lipid, we employed a multi-strategy approach combining three statistical methods. First, partial least squares discriminant analysis (PLS-DA) identified lipids with the greatest intensity discriminatory capacity at each time point. Second, mean area under the curve (AUC) analysis quantified the cumulative changes of individual lipids during recovery and identified lipids with the largest cumulative differences between intensities. Finally, two-way ANOVA detected intensity-by-time interaction effects, identifying lipids with different dynamic patterns between intensities.

PLS-DA with VIP scoring identified the 20 lipids with highest VIP scores (2.1 to 2.8) at each time point **(**Fig. 3A-C**)**. LPA18:2, PC32:2, PI36:4 (18:2_18:2), TAG58:8 (22:6), TAG46:3 (18:2), TAG46:2 (16:0), TAG46:2 (18:2), TAG48:4 (18:2), and TAG48:3 (18:1) showed high VIP scores at all three time points, with TAGs comprising 70% of these lipids.

Mean AUC analysis revealed distinct lipid profiles between groups (Fig. 3D-F), except for shared up-regulated GM3 d18:1/18:1. Further AUC analysis identified the top 20 lipids with the greatest intensity differences in cumulative response (-log₁₀ *P* 2.0 to 8.0), with PC32:2 exhibiting the most pronounced intensity-dependent changes.

Five intensity-dependent lipids were identified by integrating PLS-DA with VIP scoring (Fig. 3A-C), mean AUC analysis (Fig. 3D-F), and two-way ANOVA (Fig. 3G). These specific species, which included PC32:2, LPA18:2, TAG46:3 (18:2), TAG46:2 (18:2), and TAG48:4 (18:2), consistently emerged as significant in each analytical approach.

### Temporal dynamics of serum lipids and structural characterization of selected lipid classes

We identified three distinct lipid dynamic patterns through C-mean fuzzy cluster analysis to investigate post-swimming changes in serum lipids **(**Fig. 4A-C**)**. Cluster 1 showed persistent decline (MICT *n* = 50, HIIT *n* = 65, Fig. 4A), while clusters 2 and 3 (recovery clusters) showed decline-recovery (MICT *n* = 69, HIIT *n* = 137, Fig. 4B) and rise-recovery patterns (MICT *n* = 27, HIIT *n* = 70, Fig. 4C), respectively. HIIT regulated more lipids than MICT in all clusters. In clusters 2 and 3 (Fig. 4B-C), HIIT regulated 2.27 and 2.59 times more lipids, respectively. No co-regulated lipids between HIIT and MICT were observed in cluster 3.

At the subclass level **(**Fig. 4D**)**, HIIT regulated a greater number of lipids in nearly all subclasses across clusters, while MICT regulated more lipids only in specific subclasses such as FFA in cluster 3. TAG, the most responsive subclass to swimming, was predominantly regulated by HIIT in clusters 1 and 2 but by MICT in cluster 3. SL, PS, and HexCer were HIIT-exclusive, with no MICT-exclusive subclasses identified. MICT showed cluster-specific regulation of PA, PC, PI and their hydrolysis products (LPA, LPC, LPI), with the former appearing only in cluster 1 and the latter only in cluster 2.

To examine whether exercise intensity selectively mobilizes lipids with specific structural features, we analyzed the distribution of carbon chain length and double bond count among significantly regulated lipid species within each temporal cluster (Fig. 4E-G). For TAG **(**Fig. 4E**)**, persistently declining TAGs in cluster 1 were predominantly shorter-chain and more saturated in HIIT, but longer-chain and more unsaturated in MICT. In cluster 2, where regulatory effects were stronger, HIIT-regulated TAGs had longer chains and broader double bond count ranges than MICT-regulated TAGs. In cluster 3, both intensities showed notably weaker effects, with HIIT-regulated TAGs having longer chains and higher unsaturation.

For phospholipids **(**Fig. 4F**)**, HIIT and MICT differed primarily in subclass composition and unsaturation in cluster (1) These differences expanded to include chain length in cluster (2) cluster 3 showed intensity specificity, with only HIIT regulating phospholipids.

For sphingolipids (Fig. 4G), HIIT and MICT differed in subclass, chain length, and unsaturation in cluster 1. In clusters 2 and 3, only HIIT regulated sphingolipids, with no MICT-regulated species detected.

### Association of exercise-induced lipidome changes with intensity-dependent energy metabolism intermediates

Previous research has shown that the metabolites including TCA cycle and glycolytic intermediates, as well as certain ketones, exhibit intensity-dependent patterns^19,24^. Targeted metabolomics identified eight intensity-dependent metabolites **(**Fig. 5A**)**. To further elucidate the differential regulatory effects of swimming exercise at varying intensities on serum lipids, we performed Pearson correlation analysis between the maximal log_2_FC of all lipid molecules and the maximal log_2_FC of these eight metabolites and screened the top five lipid molecules exhibiting the highest correlation with each metabolite **(**Fig. 5B**)**.

75% of lipids were negatively correlated with intensity-dependent metabolites, with TAG and FFA as the predominant subclasses **(**Fig. 5B-C**)**. FFA showed strong negative correlations with L-lactic acid and pyruvate, sharing four identical molecules **(**Fig. 5B**)**. The top five lipids most strongly correlated with (R)-3-hydroxybutyric acid and L-malic acid were all TAGs **(**Fig. 5B**)**, with two TAGs shared between the two metabolites. Fumaric acid, as a precursor of L-malic acid, shared three negatively correlated TAGs with L-malic acid **(**Fig. 5B**)**. Analysis of the fatty acid chain composition of negatively correlated lipids shown that 18:2, 16:0, and 18:1 were the major fatty acid chain types, with 18:2 exhibiting the highest frequency (*n* = 17) **(**Fig. 5D**)**.

## Discussion

Baseline characteristics confirmed homogeneity between groups (Table 1). Combined with swimming test results (Table 2), we successfully established an acute exercise model matched for total energy expenditure but differing in intensity. While Zhang et al.^25^ found chronic moderate-intensity training altered more lipid species than vigorous training, our acute results show HIIT induced greater perturbations than MICT. This difference likely reflects acute metabolic stress versus chronic adaptations. Critically, our energy-matched design isolates intensity effects from total energy expenditure, a control absent in most prior studies. And to our knowledge, this is the first human lipidomics study to systematically compare intensity-dependent effects of acute swimming exercise on the serum lipidome, providing novel molecular insights into the early metabolic events that may contribute to long-term training adaptations.

HIIT swimming induced markedly greater lipid perturbations than MICT swimming, with downregulated lipid species reaching 2.87-fold higher levels at 30 min post-exercise (Fig. 2). This intensity-dependent metabolic response reflects the distinct neuroendocrine milieu created by high-intensity swimming. During maximal swimming efforts, catecholamine secretion activates the cAMP-protein kinase cascade that phosphorylates hormone-sensitive lipase and perilipin proteins, triggering maximal adipose tissue lipolysis^26,27^. HIIT protocols create elevated excess post-exercise oxygen consumption^28,29^ and sustained growth hormone elevation^30^ for 12–24 h post-exercise, mechanistically explaining the progressive intensification of lipid downregulation during recovery. The differential temporal dynamics of lipid upregulation between HIIT and MICT illuminate intensity-specific metabolic flux patterns. HIIT produces immediate peak upregulation followed by rapid decline, whereas MICT generates stable modest upregulation with throughout recovery^31–33^. TAG emerged as the most responsive lipid subclass to acute swimming (Fig. 2D), with HIIT downregulating more TAG species than MICT in a time-dependent manner, reaching 2.5-fold greater at 30 min post-exercise. The greater TAG downregulation observed with HIIT likely stems from its superior catecholamine response, which triggers robust PKA-mediated hormone-sensitive lipase (HSL) phosphorylation^34^. This enhanced lipolytic activation persists into the early recovery period, progressively amplifying TAG hydrolysis^32^. The time-dependent increase reflects the cumulative lipolytic effect as activated HSL continues mobilizing intramuscular and adipose TAG stores post-exercise^35^. In addition, CE and SL were specific regulated by HIIT (Fig. 2D). Given that CE responds rapidly to exercise-induced shifts in HDL and LDL metabolism^36^, its specific modulation by HIIT likely reflects more significant lipoprotein remodeling. Meanwhile, SL are involved in inflammatory signaling, apoptosis, and insulin sensitivity regulation^37,38^. Therefore, the specific modulation of SL by HIIT may indicate heightened oxidative stress, increased demands for membrane remodeling, and modulated inflammatory signaling pathways^39^.

Through multivariate statistical analysis, we identified five lipid molecules exhibiting robust intensity-dependent responses, including PC32:2, LPA18:2, TAG46:3 (18:2), TAG46:2 (18:2) and TAG48:4 (18:2) (Fig. 3). PC serves as a major membrane structural component participating in metabolic stress-induced remodeling^40^. LPA18:2, as a signaling molecule involved in inflammation, and cell proliferation^41^, demonstrated significant intensity effects despite down-regulation after both exercise modalities. The predominance of TAGs once again reflects the central role of TAG in distinguishing metabolic states induced by different exercise intensities. Beyond the absolute abundance changes, the structural pattern emerges from these intensity-sensitive lipids. We observed the recurrent presence of the 18:2 (linoleic acid) fatty acid chain across multiple key molecules. Linoleic acid is an essential omega-6 polyunsaturated fatty acid with multiple metabolic fates. One critical pathway is its conversion into arachidonic acid (AA)^42^, which serves as the substrate for synthesis of eicosanoids, including prostaglandins, leukotrienes, and thromboxanes^43^. These lipid mediators regulate physiological processes such as inflammation, immune responses, vascular function, and tissue repair^44^. Additionally, linoleic acid can be oxidized to bioactive lipid mediators such as 12,13-diHOME, which Stanford et al.^45^ identified as an exercise-induced lipokine that enhances skeletal muscle fatty acid uptake. The enrichment of 18:2-containing lipids among our intensity-dependent biomarkers suggests that linoleic acid metabolism represents a key node in intensity-specific exercise responses, potentially serving dual roles in both energy mobilization and inflammatory signaling. Given this metabolic cascade, the selective mobilization of 18:2-rich lipids likely reflect intensity-specific requirements for post-exercise signaling and recovery. We hypothesize that HIIT, which is associated with more pronounced acute inflammatory responses and tissue microdamage, may drive the rapid conversion of 18:2-rich lipids into pro-inflammatory eicosanoids^46^.

We identified three distinct lipid dynamic patterns through C-mean fuzzy cluster analysis (Fig. 4A-C). We propose a hierarchical model of metabolic mobilization composed of a basal layer of essential fuel consumption (Cluster 1), an intensity-sensitive layer reflecting homeostatic perturbation and recovery (Cluster 2), and an intensity-specific stress layer (Cluster 3). Cluster 2, where HIIT regulated nearly four times more lipids than MICT, represents the primary arena of dose-response, highlighting the metabolic disturbance and homeostatic overshoot required to recover from high-intensity exercise^47^. The complete divergence of lipids in Cluster 3 demonstrates that HIIT and MICT trigger qualitatively different metabolic pathways. The functional roles of lipid subclasses were segregated across these clusters (Fig. 4D). TAGs were robustly regulated by HIIT in Clusters 1 and 2, consistent with higher energy expenditure and greater reliance on fat oxidation during post-exercise recovery^48^. However, Cluster 3 revealed a reversal, with MICT exerting stronger effects on FFAs and certain TAGs.

Beyond these inter-subclass differences, a more granular examination of TAG molecular structures reveals additional layers of intensity-dependent selectivity. The intensity-dependent structural selectivity in TAG mobilization reveals distinct molecular preferences (Fig. 4E). Fatty acid mobilization from triacylglycerols occurs selectively based on chain length and saturation, with relative mobilization rates varying substantially across different structural types^49^. In cluster 1, HIIT preferentially mobilized shorter-chain, more saturated TAGs, whereas MICT favored longer-chain, more unsaturated species (Fig. 4E). This pattern reflects HSL substrate specificity, which exhibits marked variation in relative hydrolysis rates across fatty acid structures^50^, and is supported by a study on skiers^51^. The shorter, more saturated TAGs during HIIT likely reflects their greater accessibility at the lipid-water interface of lipid droplets during intense lipolytic stimulation^49^. The persistent decline of these TAGs reflects both enhanced consumption and suppressed production, with consumption being the dominant factor. Post-exercise fatty acid oxidation remains substantially elevated for hours, driven by increased muscle uptake, mitochondrial import, and lipoprotein lipase-mediated hydrolysis^52,53^. Isotope tracer data show that mobilization of fatty acids surpasses their oxidation during the recovery phase, creating flux that drives net TAG depletion^54,55^. Suppressed hepatic VLDL-TAG secretion and inhibited de novo lipogenesis contribute secondarily^56,57^. While TAGs represent the primary metabolic fuel mobilized during exercise, lipids also serve critical signaling and structural functions that may be differentially regulated by exercise intensity. Our data reveal an exclusive regulatory domain for HIIT over specific signaling lipids. For SL (Fig. 4D), HIIT’s biphasic regulation (Clusters 1 and 3) suggests it surpasses a critical stress threshold to activate key signaling pathways implicated in cellular stress and insulin resistance^58^. Structurally, HIIT modulated various sphingolipids with diverse chain lengths and degrees of unsaturation (Fig. 4F-G), implying activation of multiple, function-specific pathways. In contrast, MICT induced a more ordered remodeling of phospholipids, evidenced by separation of precursor lipids (Cluster 1) from their hydrolyzed lysophospholipid products (Cluster 2)(Fig. 4D), pointing towards a controlled enzymatic process^59^. This structural remodeling also showed an intensity-dependent evolution (Fig. 4F-G), expanding from initial differences in unsaturation to also include chain length, indicating that HIIT induces a deeper and more extensive membrane reorganization compared to MICT^44^.

Our correlation analysis revealed that 75% of exercise-responsive lipids exhibited negative correlations with intensity-dependent metabolites (Fig. 5B). This pattern confirms that lipid mobilization inversely relates to exercise intensity, reflecting the strategic metabolic shift from fat to carbohydrate oxidation as intensity increases^32,60^. TAG and FFA dominated the negatively correlated lipids (Fig. 5C), reflecting fundamental metabolic priorities whereby TAG serves as dedicated energy storage mobilized during exercise^61^.The strong negative correlations between FFA and glycolytic products (Fig. 5B) reflects the Randle cycle operating in reverse during intense exercise^62^. While AMPK activation during moderate exercise enhances both glucose and fatty acid oxidation^63^, high-intensity exercise suppresses FFA oxidation through declining free carnitine availability, reduced pH inhibiting carnitine palmitoyltransferase-I, and increased malonyl-CoA^33,63^. Examining specific metabolites within these correlations reveals distinct functional associations. All five lipids most strongly correlated with 3-hydroxybutyric acid were TAGs (Fig. 5B), with two molecules shared with L-malic acid correlations, indicating that these specific triglyceride species channel fatty acid-derived carbons into aerobic pathways efficiently^64^. When acetyl-CoA production from TAG-derived fatty acid β-oxidation exceeds TCA cycle capacity, ketogenesis redirects excess acetyl-CoA to 3-hydroxybutyric acid synthesis, which then provides an alternative fuel source for peripheral tissues^64,65^. Fumaric acid and L-malic acid shared three negatively correlated TAGs, suggesting these specific triglyceride species possess structural features that couple their oxidation products to this metabolic segment. This observed selectivity ultimately traces back to the constituent fatty acids within these TAG molecules. The predominance of 18:2 linoleic acid, 16:0 palmitic acid, and 18:1 oleic acid in negatively correlated lipids (Fig. 5D) reveals exercise-responsive fatty acid selectivity. Linoleic acid’s highest frequency indicates preferential mobilization of polyunsaturated fatty acids during exercise despite requiring additional β-oxidation enzymes to accommodate double bond geometry, whereas saturated palmitic acid undergoes standard β-oxidation cycles^66,67^. This apparent paradox resolves when considering that polyunsaturated fatty acids demonstrate enhanced membrane fluidity facilitating more efficient mobilization from adipose triglyceride stores and superior binding affinity to fatty acid transport proteins for cellular uptake^68,69^. Palmitic acid, though comprising 20–25% of adipose fatty acids, shows lower intensity-dependent responsiveness than its abundance would predict, likely because its saturated structure renders it less accessible to hormone-sensitive lipase during catecholamine-stimulated lipolysis^66^.

Beyond mechanistic insights, our findings have direct implications for the emerging field of precision exercise medicine. The intensity-dependent lipid biomarkers identified here hold translational potential for precision exercise medicine^70^. These molecular signatures could serve as objective intensity verification tools, particularly valuable in clinical populations where heart rate or perceived exertion may be unreliable. The structural selectivity patterns observed suggest that HIIT preferentially mobilizes shorter-chain more saturated TAGs, indicating potential for metabolic phenotyping to guide personalized intensity selection. Meanwhile, the enrichment of linoleic acid-containing lipids points toward dietary omega-6/omega-3 optimization strategies. However, clinical translation requires developing rapid, cost-effective assays; establishing population-specific reference ranges; and demonstrating associations between acute biomarker responses and long-term health outcomes^71^. As precision medicine advances, molecular biomarkers may complement traditional monitoring to enable truly personalized exercise prescriptions.

Certain limitations should be acknowledged. First, the subject population was limited to healthy young college students, which restricts generalization of results to populations of different ages, genders, and health statuses. Second, gender-specific analyses were not conducted, potentially overlooking sex differences in physiological responses to swimming exercise. Third, the observation time window in the present study was limited to 30 min post-exercise, which may have missed changes in lipid metabolism on longer time scales. Additionally, serum lipidomics mainly reflects circulating lipid status and cannot directly reveal tissue-specific lipid metabolism changes in skeletal muscle and adipose tissue, which limits in-depth understanding of tissue-specific mechanisms to some extent. And population-level results may mask significant individual differences across varying exercise intensities. Thus, future longitudinal research incorporating tissue-specific analyses, longer observation periods, and diverse populations is warranted to address these limitations and fully realize the clinical potential of exercise lipidomics.

## Conclusion

This study establishes that exercise intensity functions as an independent metabolic regulator, reorganizing lipid metabolism through mechanisms distinct from total energy expenditure. Under matched caloric conditions, high-intensity and moderate-intensity exercise elicited fundamentally divergent lipidomic responses, demonstrating that the quality of metabolic stress, not merely its magnitude, determines how the body prioritizes substrate mobilization. Intensity governs not only the breadth of lipid perturbation but also the structural selectivity of mobilized species and the temporal dynamics of post-exercise recovery, revealing a hierarchical organization of metabolic responses that operates across distinct regulatory thresholds. These findings provide a molecular foundation for intensity-stratified exercise prescription and suggest that optimizing metabolic health outcomes requires targeting specific intensity domains rather than simply accumulating exercise volume.

## Data Availability

The datasets generated during the current study are not publicly available due to privacy restrictions but are available from the corresponding author upon reasonable request and with appropriate ethical approval.

## References

[1] Dibben, G. O. et al. Exercise-based cardiac rehabilitation for coronary heart disease: a meta-analysis. *Eur. Heart J.* **44**, 452–469 (2023). 36746187 10.1093/eurheartj/ehac747 PMC9902155

[2] Kazemi, A. et al. Leisure-time and occupational physical activity and risk of cardiovascular disease incidence: a systematic-review and dose-response meta-analysis of prospective cohort studies. *Int. J. Behav. Nutr. Phys. Act.* **21**, 45 (2024). 38659024 10.1186/s12966-024-01593-8 PMC11044601

[3] Qiu, Y. et al. Exercise sustains the hallmarks of health. *J. Sport Health Sci.* **12**, 8–35 (2023). 36374766 10.1016/j.jshs.2022.10.003 PMC9923435

[4] Torma, F. et al. High intensity interval training and molecular adaptive response of skeletal muscle. *Sports Med. Health Sci.* **1**, 24–32 (2019). 35782463 10.1016/j.smhs.2019.08.003 PMC9219277

[5] MacInnis, M. J. et al. Superior mitochondrial adaptations in human skeletal muscle after interval compared to continuous single-leg cycling matched for total work. *J. Physiol.* **595**, 2955–2968 (2017). 27396440 10.1113/JP272570 PMC5407978

[6] Li, F. H. et al. Beneficial autophagic Activities, mitochondrial Function, and metabolic phenotype adaptations promoted by High-Intensity interval training in a rat model. *Front. Physiol.* **9**, 571 (2018). 29875683 10.3389/fphys.2018.00571 PMC5974531

[7] Pengam, M. et al. Moderate intensity continuous versus high intensity interval training: metabolic responses of slow and fast skeletal muscles in rat. *PLoS One*. **18**, e0292225 (2023). 37792807 10.1371/journal.pone.0292225 PMC10550171

[8] Tanaka, H. Swimming exercise: impact of aquatic exercise on cardiovascular health. *Sports Med.* **39**, 377–387 (2009). 19402742 10.2165/00007256-200939050-00004

[9] Lazar, J. M., Khanna, N., Chesler, R. & Salciccioli, L. Swimming and the heart. *Int. J. Cardiol.* **168**, 19–26 (2013). 23602872 10.1016/j.ijcard.2013.03.063

[10] Mohr, M. et al. High-Intensity Intermittent Swimming Improves Cardiovascular Health Status for Women with Mild Hypertension. Biomed Res Int. 728289 (2014). (2014). 10.1155/2014/728289 PMC4000940 24812628

[11] Klonizakis, M. & Mitropoulos, A. Assessing the effect of regular swimming exercise on the micro- and macrovascular physiology of older adults (ACELA II study). *Front. Physiol.* **14**, 1223558 (2023). 37766753 10.3389/fphys.2023.1223558 PMC10520699

[12] Smart, N. A. et al. The effect of exercise training on blood lipids: A systematic review and Meta-analysis. *Sports Med.* **55**, 67–78 (2025). 39331324 10.1007/s40279-024-02115-z PMC11787149

[13] Ivanova, P. T., Milne, S. B., Myers, D. S. & Brown, H. A. Lipidomics: a mass spectrometry based, systems level analysis of cellular lipids. *Curr. Opin. Chem. Biol.* **13**, 526–531 (2009). 19744877 10.1016/j.cbpa.2009.08.011 PMC2787871

[14] Wenk, M. R. Lipidomics: new tools and applications. *Cell* **143**, 888–895 (2010). 21145456 10.1016/j.cell.2010.11.033

[15] Salihovic, S., Lamichane, S., Hyötyläinen, T. & Orešič, M. Recent advances towards mass spectrometry-based clinical lipidomics. *Curr. Opin. Chem. Biol.* **76**, 102370 (2023). 37473482 10.1016/j.cbpa.2023.102370

[16] Sarzynski, M. A. et al. Abstract 499: Exercise Training Alters the Plasma Lipidomic Profile. *Thromb. Vascular Biology* **39**, A499–A499 (2019).

[17] Tham, Y. K. et al. Lipidomic profiles of the heart and circulation in response to exercise versus cardiac pathology: A resource of potential biomarkers and drug targets. *Cell. Rep.* **24**, 2757–2772 (2018). 30184508 10.1016/j.celrep.2018.08.017

[18] Schranner, D., Kastenmüller, G., Schönfelder, M., Römisch-Margl, W. & Wackerhage, H. Metabolite concentration changes in humans after a bout of exercise: a systematic review of exercise metabolomics studies. *Sports Med. Open.* **6**, 11 (2020). 32040782 10.1186/s40798-020-0238-4 PMC7010904

[19] Tang, C. et al. Dynamic metabolic changes driven by Exercise-Intensity in acute swimming. *Med. Sci. Sports Exerc.*10.1249/MSS.0000000000003800 (2025). 10.1249/MSS.0000000000003800 PMC12893147 40601479

[20] Miao, H. et al. Lipidome atlas of the developing heart uncovers dynamic membrane lipid attributes underlying cardiac structural and metabolic maturation. Research 2022, 0006 (2022). 10.34133/research.0006 PMC11407523 39290970

[21] Zimmermann, M., Sauer, U. & Zamboni, N. Quantification and mass isotopomer profiling of α-keto acids in central carbon metabolism. *Anal. Chem.* **86**, 3232–3237 (2014). 24533614 10.1021/ac500472c

[22] Lam, S. M. et al. A multi-omics investigation of the composition and function of extracellular vesicles along the Temporal trajectory of COVID-19. *Nat. Metab.* **3**, 909–922 (2021). 34158670 10.1038/s42255-021-00425-4

[23] Morville, T., Sahl, R. E., Moritz, T., Helge, J. W. & Clemmensen, C. Plasma metabolome profiling of resistance exercise and endurance exercise in humans. *Cell. Rep.* **33**, 108554 (2020). 33378671 10.1016/j.celrep.2020.108554

[24] Wu, B. et al. Sexual dimorphism in the serum metabolome following acute exhaustive exercise. *Biol. Sex. Differ.* **16**, 91 (2025). 41199406 10.1186/s13293-025-00780-x PMC12593952

[25] Zhang, Y. et al. Moderate-intensity exercise training uniquely modulates Circulating lipid species beyond classical lipid levels in humans. *eBioMedicine* **118**, 105849 (2025). 40669298 10.1016/j.ebiom.2025.105849 PMC12283555

[26] Nielsen, T. S., Jessen, N., Jørgensen, J. O. L., Møller, N. & Lund, S. Dissecting adipose tissue lipolysis: molecular regulation and implications for metabolic disease. *J. Mol. Endocrinol.* **52**, R199–222 (2014). 24577718 10.1530/JME-13-0277

[27] Zechner, R. et al. FAT SIGNALS - Lipases and lipolysis in lipid metabolism and signaling. *Cell. Metab.* **15**, 279–291 (2012). 22405066 10.1016/j.cmet.2011.12.018 PMC3314979

[28] Jiang, L., Zhang, Y., Wang, Z. & Wang, Y. Acute interval running induces greater excess post-exercise oxygen consumption and lipid oxidation than isocaloric continuous running in men with obesity. *Sci. Rep.* **14**, 9178 (2024). 38649759 10.1038/s41598-024-59893-9 PMC11035584

[29] Børsheim, E. & Bahr, R. Effect of exercise intensity, duration and mode on post-exercise oxygen consumption. *Sports Med.* **33**, 1037–1060 (2003). 14599232 10.2165/00007256-200333140-00002

[30] Pritzlaff, C. J. et al. Impact of acute exercise intensity on pulsatile growth hormone release in men. J Appl Physiol 87, 498–504 (1999). (1985). 10.1152/jappl.1999.87.2.498 10444604

[31] Horowitz, J. F., Mora-Rodriguez, R., Byerley, L. O. & Coyle, E. F. Lipolytic suppression following carbohydrate ingestion limits fat oxidation during exercise. *Am. J. Physiol.* **273**, E768–775 (1997). 9357807 10.1152/ajpendo.1997.273.4.E768

[32] Romijn, J. A. et al. Regulation of endogenous fat and carbohydrate metabolism in relation to exercise intensity and duration. *Am. J. Physiol.* **265**, E380–391 (1993). 8214047 10.1152/ajpendo.1993.265.3.E380

[33] van Loon, L. J. C., Greenhaff, P. L., Constantin-Teodosiu, D., Saris, W. H. M. & Wagenmakers, A. J. M. The effects of increasing exercise intensity on muscle fuel utilisation in humans. *J. Physiol.* **536**, 295–304 (2001). 11579177 10.1111/j.1469-7793.2001.00295.x PMC2278845

[34] Liu, Y. et al. Post-exercise effects and Long-Term training adaptations of hormone sensitive lipase lipolysis induced by High-Intensity interval training in adipose tissue of mice. *Front. Physiol.* **11**, 535722 (2020). 33324231 10.3389/fphys.2020.535722 PMC7723847

[35] Neuendorf, T., Kaden, M., Wachler, E., Schumann, M. & Nitzsche, N. Metabolic responses of eccentric accentuated flywheel squats by a Multi-set protocol with increasing loads. *J. Sci. Sport Exerc.* **7**, 422–429 (2025).

[36] Stanton, K. M. et al. Moderate- and High‐Intensity exercise improves lipoprotein profile and cholesterol efflux capacity in healthy young men. *J. Am. Heart Assoc.* **11**, e023386 (2022). 35699182 10.1161/JAHA.121.023386 PMC9238648

[37] Shepherd, S. O. et al. Lipid droplet remodelling and reduced muscle ceramides following sprint interval and moderate-intensity continuous exercise training in obese males. *Int. J. Obes. (Lond)*. **41**, 1745–1754 (2017). 28736444 10.1038/ijo.2017.170

[38] Bergman, B. C. et al. Serum sphingolipids: relationships to insulin sensitivity and changes with exercise in humans. *Am. J. Physiol. Endocrinol. Metab.* **309**, E398–E408 (2015). 26126684 10.1152/ajpendo.00134.2015 PMC4537923

[39] Mallett, G. S. & McGrath, K. Effect of endurance exercise on markers of oxidative stress: A systematic review. *J. SCI. SPORT Exerc.* **7**, 175–196 (2025).

[40] Jäger, R., Purpura, M. & Kingsley, M. Phospholipids and sports performance. *J. Int. Soc. Sports Nutr.* **4**, 5 (2007). 17908342 10.1186/1550-2783-4-5 PMC1997116

[41] Lee, J. H., Kim, D., Oh, Y. S. & Jun, H. S. Lysophosphatidic acid signaling in diabetic nephropathy. *Int. J. Mol. Sci.* **20**, 2850 (2019). 31212704 10.3390/ijms20112850 PMC6600156

[42] Serhan, C. N. & Levy, B. D. Resolvins in inflammation: emergence of the pro-resolving superfamily of mediators. *J. Clin. Invest.* **128**, 2657–2669 (2018). 10.1172/JCI97943 PMC6025982 29757195

[43] Dennis, E. A. & Norris, P. C. Eicosanoid storm in infection and inflammation. *Nat. Rev. Immunol.* **15**, 511–523 (2015). 26139350 10.1038/nri3859 PMC4606863

[44] Nieman, D. C. & Wentz, L. M. The compelling link between physical activity and the body’s defense system. *J. Sport Health Sci.* **8**, 201–217 (2019). 31193280 10.1016/j.jshs.2018.09.009 PMC6523821

[45] Stanford, K. I. et al. 12,13-diHOME: an Exercise-Induced lipokine that increases skeletal muscle fatty acid uptake. *Cell. Metab.* **27**, 1111–1120e3 (2018). 29719226 10.1016/j.cmet.2018.03.020 PMC5935136

[46] Peake, J. M., Neubauer, O., Walsh, N. P. & Simpson, R. J. Recovery of the immune system after exercise. J Appl Physiol 122, 1077–1087 (2017). (1985). 10.1152/japplphysiol.00622.2016 27909225

[47] Egan, B. & Zierath, J. R. Exercise metabolism and the molecular regulation of skeletal muscle adaptation. *Cell. Metab.* **17**, 162–184 (2013). 23395166 10.1016/j.cmet.2012.12.012

[48] San-Millán, I. & Brooks, G. A. Assessment of metabolic flexibility by means of measuring blood Lactate, Fat, and carbohydrate oxidation responses to exercise in professional endurance athletes and Less-Fit individuals. *Sports Med.* **48**, 467–479 (2018). 28623613 10.1007/s40279-017-0751-x

[49] Raclot, T. Selective mobilization of fatty acids from adipose tissue triacylglycerols. *Prog Lipid Res.* **42**, 257–288 (2003). 12689620 10.1016/s0163-7827(02)00066-8

[50] Raclot, T., Holm, C. & Langin, D. Fatty acid specificity of hormone-sensitive lipase. Implication in the selective hydrolysis of triacylglycerols. *J. Lipid Res.* **42**, 2049–2057 (2001). 11734578

[51] Lyudinina, A. Y., Ivankova, G. E. & Bojko, E. R. Priority use of medium-chain fatty acids during high-intensity exercise in cross-country skiers. *J. Int. Soc. Sports Nutr.* **15**, 57 (2018). 30526607 10.1186/s12970-018-0265-4 PMC6288941

[52] Henderson, G. C. et al. Lipolysis and fatty acid metabolism in men and women during the postexercise recovery period. *J. Physiol.* **584**, 963–981 (2007). 17855762 10.1113/jphysiol.2007.137331 PMC2277001

[53] Lundsgaard, A. M., Fritzen, A. M. & Kiens, B. The importance of fatty acids as nutrients during Post-Exercise recovery. *Nutrients* **12**, 280 (2020). 31973165 10.3390/nu12020280 PMC7070550

[54] Horowitz, J. F. Fatty acid mobilization from adipose tissue during exercise. *Trends Endocrinol. Metab.* **14**, 386–392 (2003). 14516937 10.1016/s1043-2760(03)00143-7

[55] Bahr, R., Hansson, P. & Sejersted, O. M. Triglyceride/fatty acid cycling is increased after exercise. *Metabolism* **39**, 993–999 (1990). 2392063 10.1016/0026-0495(90)90313-2

[56] Schenk, S. & Horowitz, J. F. Acute exercise increases triglyceride synthesis in skeletal muscle and prevents fatty acid–induced insulin resistance. *J. Clin. Invest.* **117**, 1690–1698 (2007). 17510709 10.1172/JCI30566 PMC1866251

[57] Gemmink, A., Schrauwen, P. & Hesselink, M. K. C. Exercising your fat (metabolism) into shape: a muscle-centred view. *Diabetologia* **63**, 1453–1463 (2020). 32529413 10.1007/s00125-020-05170-z PMC7351830

[58] Petersen, M. C. & Shulman, G. I. Mechanisms of insulin action and insulin resistance. *Physiol. Rev.* **98**, 2133–2223 (2018). 30067154 10.1152/physrev.00063.2017 PMC6170977

[59] Dennis, E. A., Cao, J., Hsu, Y. H., Magrioti, V. & Kokotos, G. Phospholipase A2 enzymes: physical Structure, biological Function, disease Implication, chemical Inhibition, and therapeutic intervention. *Chem. Rev.* **111**, 6130–6185 (2011). 21910409 10.1021/cr200085w PMC3196595

[60] Brooks, G. A. & Mercier, J. Balance of carbohydrate and lipid utilization during exercise: the ‘crossover’ concept. J Appl Physiol 76, 2253–2261 (1994). (1985). 10.1152/jappl.1994.76.6.2253 7928844

[61] Kelley, D. E., Mokan, M., Simoneau, J. A. & Mandarino, L. J. Interaction between glucose and free fatty acid metabolism in human skeletal muscle. *J. Clin. Invest.* **92**, 91–98 (1993). 8326021 10.1172/JCI116603 PMC293539

[62] Randle, P. J., Garland, P. B., Hales, C. N. & Newsholme, E. A. The glucose fatty-acid cycle. Its role in insulin sensitivity and the metabolic disturbances of diabetes mellitus. *Lancet* **1**, 785–789 (1963). 13990765 10.1016/s0140-6736(63)91500-9

[63] Hue, L. & Taegtmeyer, H. The Randle cycle revisited: a new head for an old hat. *Am. J. Physiol. Endocrinol. Metab.* **297**, E578–E591 (2009). 19531645 10.1152/ajpendo.00093.2009 PMC2739696

[64] Dubé, J. J. et al. Adipose triglyceride lipase deletion from adipocytes, but not skeletal myocytes, impairs acute exercise performance in mice. *Am. J. Physiol. Endocrinol. Metab.* **308**, E879–E890 (2015). 25783895 10.1152/ajpendo.00530.2014 PMC4436997

[65] Cox, P. J. et al. Nutritional ketosis alters fuel preference and thereby endurance performance in athletes. *Cell. Metab.* **24**, 256–268 (2016). 27475046 10.1016/j.cmet.2016.07.010

[66] Puchalska, P. & Crawford, P. A. Multi-dimensional roles of ketone bodies in fuel metabolism, signaling, and therapeutics. *Cell. Metab.* **25**, 262–284 (2017). 28178565 10.1016/j.cmet.2016.12.022 PMC5313038

[67] Watt, M. J. & Spriet, L. L. Triacylglycerol lipases and metabolic control: implications for health and disease. *Am. J. Physiol. Endocrinol. Metab.* **299**, E162–168 (2010). 20071561 10.1152/ajpendo.00698.2009

[68] Jeukendrup, A. E., Saris, W. H. & Wagenmakers, A. J. Fat metabolism during exercise: a review. Part I: fatty acid mobilization and muscle metabolism. *Int. J. Sports Med.* **19**, 231–244 (1998). 9657362 10.1055/s-2007-971911

[69] Kiens, B. Skeletal muscle lipid metabolism in exercise and insulin resistance. *Physiol. Rev.* **86**, 205–243 (2006). 16371598 10.1152/physrev.00023.2004

[70] DiMenna, F. J. & Arad, A. D. Exercise as ‘precision medicine’ for insulin resistance and its progression to type 2 diabetes: a research review. *BMC Sports Sci. Med. Rehabil*. **10**, 21 (2018). 30479775 10.1186/s13102-018-0110-8 PMC6251139

[71] Haller, N. et al. Blood-Based biomarkers for managing workload in athletes: perspectives for research on emerging biomarkers. *Sports Med.* **53**, 2039–2053 (2023). 37341908 10.1007/s40279-023-01866-5 PMC10587296

